# Phase quantification using deep neural network processing of XRD patterns

**DOI:** 10.1107/S2052252524006766

**Published:** 2024-08-12

**Authors:** Titouan Simonnet, Sylvain Grangeon, Francis Claret, Nicolas Maubec, Mame Diarra Fall, Rachid Harba, Bruno Galerne

**Affiliations:** a Institut Denis Poisson, Université d’Orléans, Université de Tours, CNRS, France; b BRGM, 45060Orléans, France; c Laboratoire PRISME, Université d’Orléans, INSA Centre Val de Loire, France; d Institut Universitaire de France (IUF) France; Tsinghua University, China

**Keywords:** composite materials, computational modeling, deep neural network, powder X-ray diffraction, calcite, gibbsite, dolomite, hematite

## Abstract

Mineral identification and quantification are key to the understanding and, hence, the capacity to predict material properties. The method of choice for mineral quantification is powder X-ray diffraction (XRD), generally using a Rietveld refinement approach. However, a successful Rietveld refinement requires preliminary identification of the phases that make up the sample. This is generally carried out manually, and this task becomes extremely long or virtually impossible in the case of very large datasets such as those from synchrotron X-ray diffraction computed tomography. To circumvent this issue, this article proposes a novel neural network (NN) method for automating phase identification and quantification. An XRD pattern calculation code was used to generate large datasets of synthetic data that are used to train the NN. This approach offers significant advantages, including the ability to construct databases with a substantial number of XRD patterns and the introduction of extensive variability into these patterns. To enhance the performance of the NN, a specifically designed loss function for proportion inference was employed during the training process, offering improved efficiency and stability compared with traditional functions. The NN, trained exclusively with synthetic data, proved its ability to identify and quantify mineral phases on synthetic and real XRD patterns. Trained NN errors were equal to 0.5% for phase quantification on the synthetic test set, and 6% on the experimental data, in a system containing four phases of contrasting crystal structures (calcite, gibbsite, dolomite and hematite). The proposed method is freely available on GitHub and allows for major advances since it can be applied to any dataset, regardless of the mineral phases present.

## Abstract 2

A deep neural network approach to the identification and quantification of powder X-ray diffraction patterns was applied and proved successful for the quantitative description of complex mineralogical assemblages consisting of up to four minerals with different structures, including different space groups, for which data augmentation is not straightforward.

## 1. Introduction

Minerals are the inorganic building blocks of soils, rocks and engineered solids (*e.g.* cement based materials). Amongst others, minerals play a vital role in human welfare (Smith, 1999 ▸), climate change mitigation and green technologies (Vidal *et al.*, 2013 ▸), remediation of pollution (Grangeon *et al.*, 2020 ▸), nuclear waste disposal (Bildstein *et al.*, 2019 ▸), CO_2_ geological storage (Bourg *et al.*, 2015 ▸), geothermal systems (Bird & Spieler, 2004 ▸), and in the understanding of planet structure and evolution (Elkins-Tanton & Seager, 2008 ▸). Understanding the occurrence, stability and evolution of mineralogical assemblages in the above-mentioned applications requires the identification of the different minerals present, deciphering their fundamental characteristics such as crystal structure (Krivovichev *et al.*, 2022 ▸) and chemistry, and the relative abundance of each mineral in the assemblage. Although crystal structure and chemical composition play a role in the intrinsic properties of minerals [*e.g.* thermal conductivity (Ndlovu *et al.*, 2011 ▸) or optical properties (Wood & Strens, 1979 ▸)], the mineral mass fraction itself is a very important parameter that controls material properties such as unconfined compressive strength (Bourg, 2015 ▸); or sorption capacity of, for example, metals or nutrients (Payne *et al.*, 2002 ▸). Since 1912 and the discovery of X-ray diffraction (XRD) by Max von Laue (Fernandez-Diaz & Lemée-Cailleau, 2013 ▸), powder XRD (XRD) has been applied to decipher crystal structures, and to identify minerals and quantify their relative proportions in an assemblage (Bish & Post, 1990 ▸). The challenging task of quantifying the different minerals in a powder has benefited from the early work of Rietveld (1969 ▸), who introduced a refinement method now commonly referred to as ‘Rietveld refinement’. This method is still regarded as state-of-the-art for quantitative analysis of XRD patterns. However, since it is based on a point-by-point error minimization using an iterative refinement method, it generally requires that a preliminary identification of mineral phases is performed before pattern modeling. Hence, in the analysis of very large datasets containing patterns with contrasting mineralogical composition, its use becomes extremely time-consuming due to the need for preliminary manual phase identification. This type of very large dataset is expected to be increasingly collected with the development of methods such as XRD computed tomography (XRD-CT) (Jacques *et al.*, 2013 ▸; Jensen *et al.*, 2015 ▸), whereby the data typically contain hundreds of thousands of patterns per slide, with a thickness equal to the beam size (Claret *et al.*, 2018 ▸). To circumvent the problems of Rietveld refinement and the processing of large datasets, deep learning (DL) methods have emerged as a promising alternative (Feng *et al.*, 2019 ▸).

In recent years, the use of DL (Goodfellow *et al.*, 2016 ▸) and neural network (NN) methods has seen considerable growth in a wide range of applications. The domain of XRD data analysis is no exception, as shown by the increasing integration of DL techniques. One can refer to Surdu & Győrgy (2023 ▸) for a comprehensive review of XRD analysis methods that employ machine learning.

XRD data analysis with machine learning first appeared in the work by Griffen (1999 ▸), with the use of artificial NNs to tackle the challenge of quantitative phase analysis of clay minerals via powder XRD. With the continuous advances in DL, the last few years have seen a significant advance in research in this domain. Though many studies have focused on classification problems, such as categorizing materials into symmetry space groups, crystal systems or extinction groups (Park *et al.*, 2017 ▸; Vecsei *et al.*, 2019 ▸; Zaloga *et al.*, 2020 ▸; Oviedo *et al.*, 2019 ▸), notable breakthroughs have been made. For example, *PQ-Net* (Dong *et al.*, 2021 ▸) introduced predictions for lattice parameters, scale factors and crystallite sizes, enriching the capabilities of XRD analysis. Recent research efforts have also been made for predicting phase fractions within multiphase compounds. Some approaches have transformed this challenge into a classification problem, dividing the output space into abundance classes (Lee *et al.*, 2020 ▸). Alternatively, some methods leverage convolutional neural networks (CNNs) for phase identification and subsequently use machine learning techniques for precise phase quantification (Lee *et al.*, 2021 ▸).

Though DL seems promising for XRD data analysis, its use remains challenging. Indeed, many DL methods require the use of large experimental datasets to be trained. This requirement is virtually impossible if the aim is to apply DL methods to natural samples such as soil. Indeed, this would require collecting thousands of patterns of mineralogical assemblages, but also, and more challenging, of pure minerals with a large yet systematic variation in lattice parameters, crystallite sizes, shapes *etc.* so as to be representative, for each mineral, of the variability that can be encountered in natural systems. These requirements would be extremely time-consuming, and would require that a collection of minerals with the necessary variation in crystallographic parameters and morphology exists. Both are hard to achieve experimentally due to the time required, but also, and more importantly, because collecting samples of all existing minerals – with a sufficient range of variation in the amount and crystal chemistry of each mineral in individual samples to account for the natural variability in chemistry, lattice parameters and morphology – is probably impossible. Alternatively, some researchers have explored data augmentation approaches (Oviedo *et al.*, 2019 ▸; Wang *et al.*, 2020 ▸). However, applicability of this method in the case of minerals that crystallize in low-symmetry space groups, with hence complicated variation in peak position as a function of changes in lattice parameters or angles, is unclear.

To circumvent this difficulty, we propose here to use synthetic data generated from crystallographic information files (Hall *et al.*, 1991 ▸). This approach has the advantage of being applicable to a wide range of multiphase compounds. It also offers the possibility to generate as much data as required for the training (up to 100 000 in this study), but also to allow for more flexibility with regards to the instruments used for actual sample analysis: with our approach, the pure diffraction profile is only calculated once, then corrected for absorption phenomena, and finally convoluted with a wavelength function.

Specifically, in the following, we detail an approach based on a CNN designed to identify and quantify phases in a multiphase material. The CNN is exclusively trained with synthetic data, and uses a loss specifically designed for proportion inference. This loss function incorporates a Dirichlet modeling approach (Sensoy *et al.*, 2018 ▸) which has been demonstrated to outperform traditional loss functions such as mean squared error (MSE) (Simonnet *et al.*, 2023 ▸). Our results demonstrate that the method performs very well on synthetic data, but also on experimental XRD patterns.

## 2. Materials and methods

### 2.1. Collection of experimental XRD patterns

XRD patterns were acquired on micronized powders using a Bruker D8 Advance diffractometer equipped with a LynxEye XE-T detector and a Cu anode (λ = 1.5418 Å). The proportions of each phase were quantified by successive weightings. Data were collected in a continuous scan mode, averaged every 0.03° 2θ, and modeled with the *Profex* software (Doebelin & Kleeberg, 2015 ▸), which is a graphical user interface to the *BGMN* software (Bergmann *et al.*, 1998 ▸). The aim of such quantitative modeling was to determine the accuracy of the Rietveld refinement on our samples, and was used for comparison with results from our CNN approach. Table 1 ▸ gives the composition of these experimental XRD patterns, determined from the weighting of each individual phase in each sample. It can thus be assumed to be the exact mineralogical composition of our samples.

### 2.2. Calculation of powder XRD patterns

XRD refers to the elastic scattering of photoelectrons from an X-ray beam by a solid. The intensity of the scattered beam is usually measured as a function of the scattering angle θ. It essentially depends on four main components: an atomic scattering factor, an interference function, a structure factor (Bish & Post, 1990 ▸) and a polarization factor. The first three components can be calculated from the knowledge of the unit-cell symmetry, size and composition (and its number of repetitions). The polarization factor is dependent on the nature of the X-ray source (*e.g.* laboratory or synchrotron source). All these factors are presented in the following and the method to generate XRD patterns is then described.

#### 2.2.1. Atomic scattering factor

The atomic scattering factor reflects the interaction of the X-rays with atoms. This interaction occurs at the electron cloud level, and the diffracted intensity increases with the number of electrons. The diffracted intensity for an angle θ = 0° corresponds to the number of electrons of the atom, and then decreases as the angle increases up to θ = 90°. Another parameter influences the intensity diffracted by an atom. This is the thermal agitation, which, in the present study, is accounted for by the Debye–Waller factor (isotropic agitation factor). This factor has the effect of attenuating intensity, particularly at high θ angles.

#### 2.2.2. Interference function

The interference function depends on the dimension and geometry of the lattice, as well as on the structural disorder. It dictates the scattering angles at which intensity can be observed. In the case of a defect-free three-dimensional-ordered crystal, the scattering angles at which diffracted intensity can be observed obey Bragg’s law: 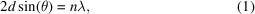
where θ is the scattering angle; *n* is a non-null integer; λ is the wavelength of the incident beam; and *d* is defined, for any crystal symmetry, as
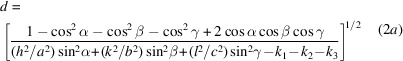
where
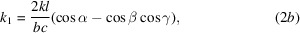

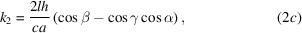

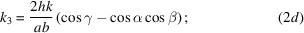
and *a*, *b* and *c* are the norms of the lattice vectors; α, β and γ are the crystallographic angles; and *h*, *k* and *l* are the Miller indices.

The width of a given reflection depends on several factors, the main one being crystallite size (*i.e.* the size of the diffracting object, which is usually smaller than the crystal due to strains or defects), while the shape (*e.g.* degree of asymmetry) depends on factors such as structural disorder (*e.g.* structural strains, stacking defects, interstratification). In this study, crystallites were assumed to have an isotropic shape, and the variation in crystallite size was modeled by a variation in the full width at half-maximum (FWHM) of Gaussian-shaped peaks.

#### 2.2.3. Structure factor

The structure factor is a continuous function of the scattering angle. It depends on the nature, position and site occupancy of each unique atom in the unit cell, and has been computed for each θ step following
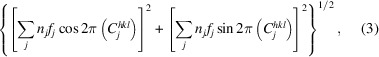
where 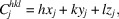
and *j* is the number of independent atomic positions; *n*_*j*_ is the occupancy of a given site; *f*_*j*_ is the atomic scattering factor of the atom occupying the *j*th position; *h*, *k* and *l* are the integration coordinates in reciprocal space; and *x*_*j*_, *y*_*j*_ and *z*_*j*_ are the fractional coordinates of the *j*th atom in the unit cell.

#### 2.2.4. Polarization factor

The polarization factor accounts for the polarization of an incident photoelectron after its interaction with matter. The polarization of the scattered photoelectron depends on that of the incident photoelectron and on the scattering angle.

#### 2.2.5. Instrumental effects

To the various components introduced in Sections 2.2.1, 2.2.2 and 2.2.3, instrumental factors were added to account for the geometry of the experiment, namely the wavelength function and the attenuation factor. The wavelength function quantitatively accounts for several instrumental factors, including the various X-ray wavelengths emitted by the anode. This function was calculated using the *Profex* (Doebelin & Kleeberg, 2015 ▸) interface to *BGMN* (Bergmann *et al.*, 1998 ▸). Adsorption of part of the incident X-rays by the solid was accounted for by calculating the X-ray mass attenuation factor at 8.0415 keV (λ_mean_ = 1.5418 Å).

### 2.3. A convolutional neural network method

**Figure 1:**
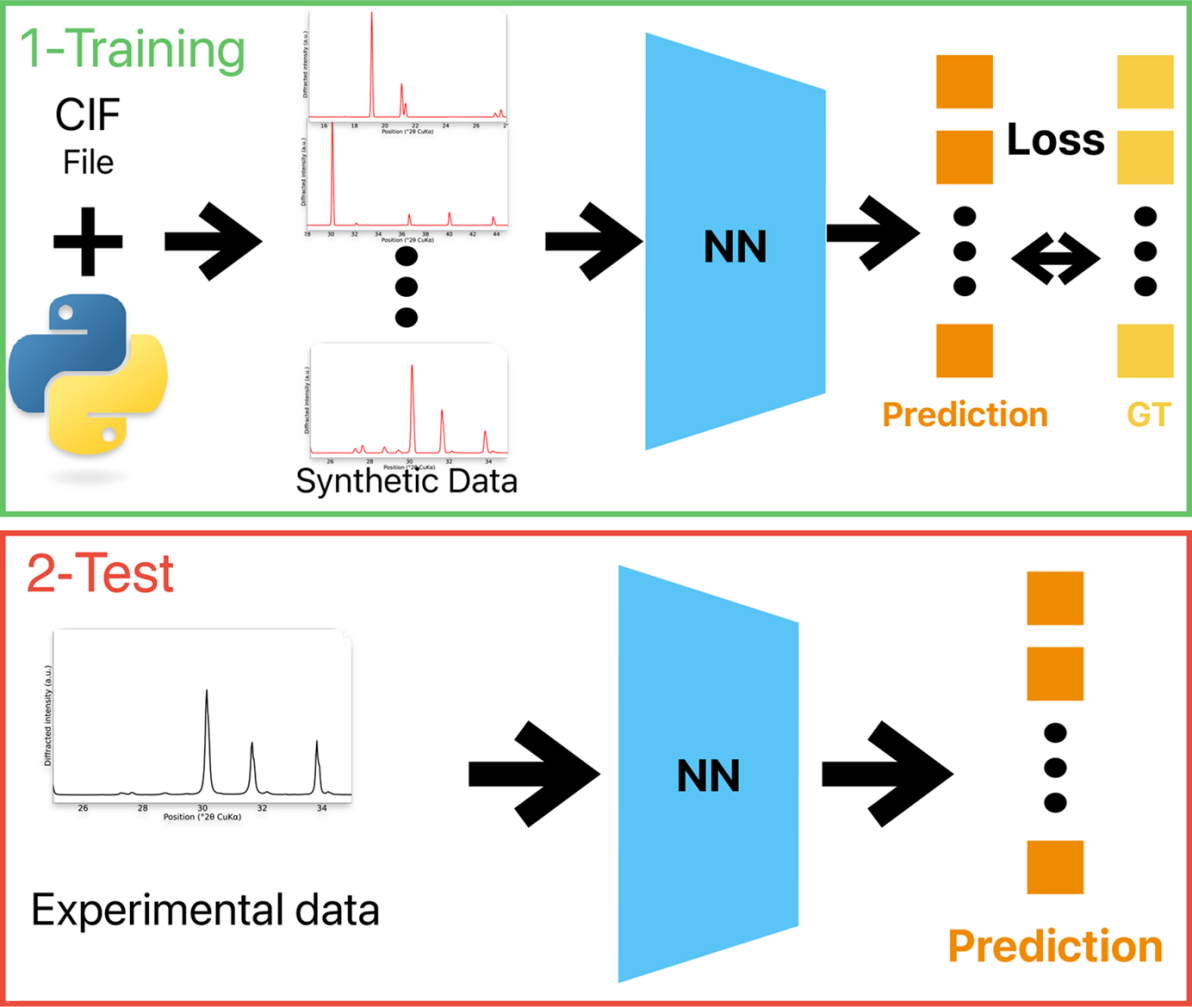
Graphical summary. First, the NN is trained to retrieve the phase proportions from XRD patterns using a synthetic database. Then the trained NN is tested with experimental XRD patterns.

The proposed NN takes an XRD pattern as input (collected on a sample that is a mixture of *K* mineral phases) and outputs an estimation of the proportion of each of these phases. More precisely, these patterns are approximated as linear combinations of *K* mineral phases (or components), thus represented as 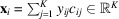, where 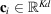 is the matrix containing the single-phase XRD patterns, and 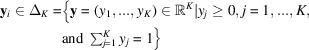
represents the *K*-dimensional simplex, that is, the set of proportion vectors. Here we consider a problem with *K* = 4 components to align with the experimental data we collected. The schematic in Fig. 1 ▸ provides an overview of the entire method, which is further described in the following.

#### 2.3.1. Neural network modeling

**Figure 2:**
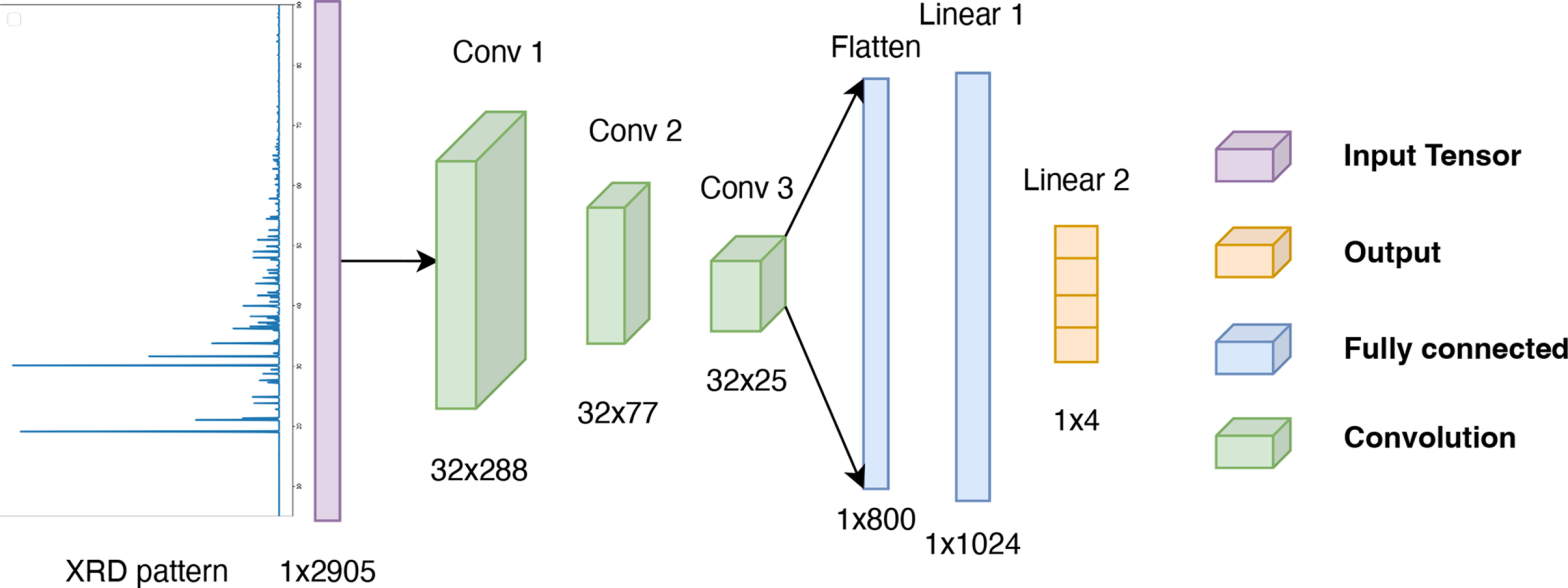
CNN architecture used to infer XRD pattern proportion (Oviedo *et al.*, 2019 ▸). The convolution kernel widths are 8, 5 and 3, respectively, and the stride values are equal to the kernel widths.

A CNN, denoted *f*, was trained and adapted from Oviedo *et al.* (2019 ▸). In this previous work, the CNN was used to classify XRD patterns according to their space group or crystallographic dimensionality. Fig. 2 ▸ describes the architecture of the CNN. It starts with three convolutional layers to extract the key signal features. The convolution kernel widths are 8, 5 and 3, respectively, and the stride values are equal to the kernel widths. Next, two linear layers are used to reduce the dimension to *K*. Each hidden layer has a ReLU activation function. Specifically, given an array **a**, 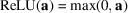, where the max operator is applied to each element. Each of the CNN layers, or operations, is associated with a large number of parameters. The NN with the parameter **θ** is denoted *f*(**x**_i_|**θ**). The architecture described above has 832 868 parameters, and these parameters are optimized during the training phase. The CNN was trained on 100 epochs with the Adam optimizer (Kingma & Ba, 2014 ▸), with a constant learning rate equal to 0.001. Five training runs were carried out to assess the stability of the method. To ensure a meaningful comparison, the same five random weight initializations were used for both databases. Since the primary aim of this study is to introduce a CNN based method for phase quantification using synthetic data and to evaluate the impact of instrumental effects, we did not investigate for the optimal values for training (number of epochs, Adam parameters *etc.*).

Finally, the loss function is a key point of the proposed method. Indeed, it was chosen to use a specifically designed loss for proportion inference using a Dirichlet model (Simonnet *et al.*, 2023 ▸). This approach proved to be more effective than alternative loss functions such as MSE or cross-entropy. It also demonstrated good stability when applied to different types of data.

This modeling approach allowed us to infer proportions by minimizing the disparity between the actual distribution of proportion and a Dirichlet distribution parameterized by an NN. The probability density function (PDF) of the Dirichlet distribution is defined as 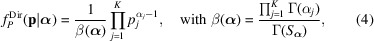
where **α** is the parameter vector, with α_*i*_ > 0 for all *i* ∈ 1, …, *K*, and *S*_**α**_ = 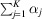 is the Dirichlet strength.

A multivariate random variable **P**_*i*_ was associated to each of the input XRD patterns **x**_*i*_ following a Dirichlet distribution: 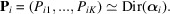
An NN was used to parameterize the Dirichlet distribution, that is using the output of the NN denoted by 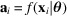 to determine the parameter vector **α**_*i*_. Due to the constraints imposed on **α**_*i*_, a transformation function, denoted ϕ, was applied to the vector **a** to ensure that all its elements were strictly positive. ϕ is a two-term function defined as follows: 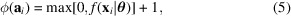
where the max operator is applied element-wise.

Once the vector **α**_*i*_ is determined (that is, after the NN training), a prediction vector 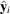 can be proposed for the proportion by considering the expectation of the parameterized Dirichlet distribution: 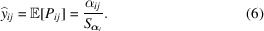
The last step is to present the function for optimizing the NN parameters **θ** to minimize the difference between the true proportion distribution and the Dirichlet distribution. This is done by minimizing the expected square error (SE): 
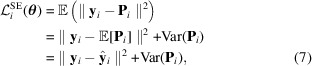
where Var**P**_*i*_ = 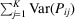 = 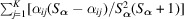.

#### 2.3.2. Synthetic database

The CNN training phase requires a substantial database to include a wide variety of XRD patterns for different mineral phases.

Using the methods introduced in Section 2.2, a database containing four different pure minerals, namely calcite [CaCO_3_ (Markgraf & Reeder, 1985 ▸)], dolomite [CaMgC_2_O_6_ (Steinfink & Sans, 1959 ▸)], gibbsite [AlO_3_H_3_ (Balan *et al.*, 2006 ▸)] and hematite [Fe_2_O_3_ (Blake *et al.*, 1966 ▸)] was built. In total, 1500 XRD patterns for each mineral were generated by varying the norm of the lattice vectors, the Debye–Waller coefficient and the FWHM. These 6000 XRD patterns will be hereafter referred to as ‘single-phase’ patterns. For each mineral, these data were divided as follows: 1000 for training, 250 for validation and the last 250 for testing, to ensure that the three resulting datasets were well separated.

Regarding the training set, from the 4000 single-phase XRD patterns (1000 for each mineral), 10 000 XRD synthetic patterns of mixtures were created by combining one to four of the different mineral phases with a given proportion vector. Similarly, a validation set and a test set were simulated, each containing 2500 XRD patterns.

**Figure 3:**
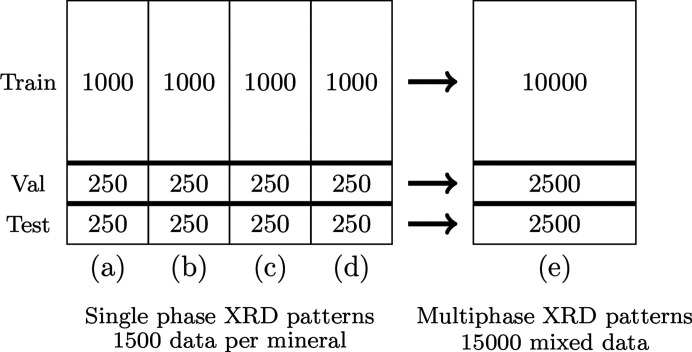
Construction of the multiphase synthetic XRD patterns database. (*a*)–(*d*) Single phase (calcite, dolomite, gibbsite and hematite); and (*e*) linear combinations of (*a*), (*b*), (*c*), (*d*) representing the multiphase patterns.

**Figure 4:**
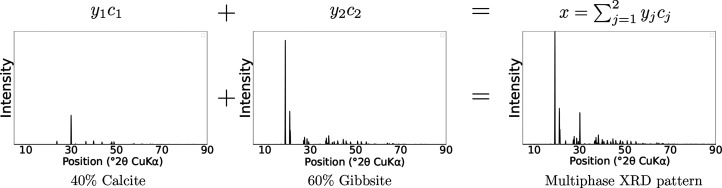
Example of a two-phase synthetic XRD pattern (40% calcite and 60% gibbsite) construction using a linear combination of two single-phase XRD patterns.

Fig. 3 ▸ summarizes the different steps of the databases construction. An example of a synthetic XRD pattern with *K* = 2 mineral phases is displayed in Fig. 4 ▸. Two different simulation schemes were tested for simulating the ‘single-phase’ patterns:

(1) Database without instrumental effects (Dw/oIE). This dataset only includes the data resulting from the calculation of pure XRD patterns, without any instrumental effects.

(2) Database with instrumental effects (DwIE). This second database contains data including the instrumental effects discussed in Section 2.2.5 (*i.e.* wavelength function and attenuation factor). The evaluation of the database on experimental data is completed by a pre-processing treatment to remove a background of constant value.

**Figure 5:**
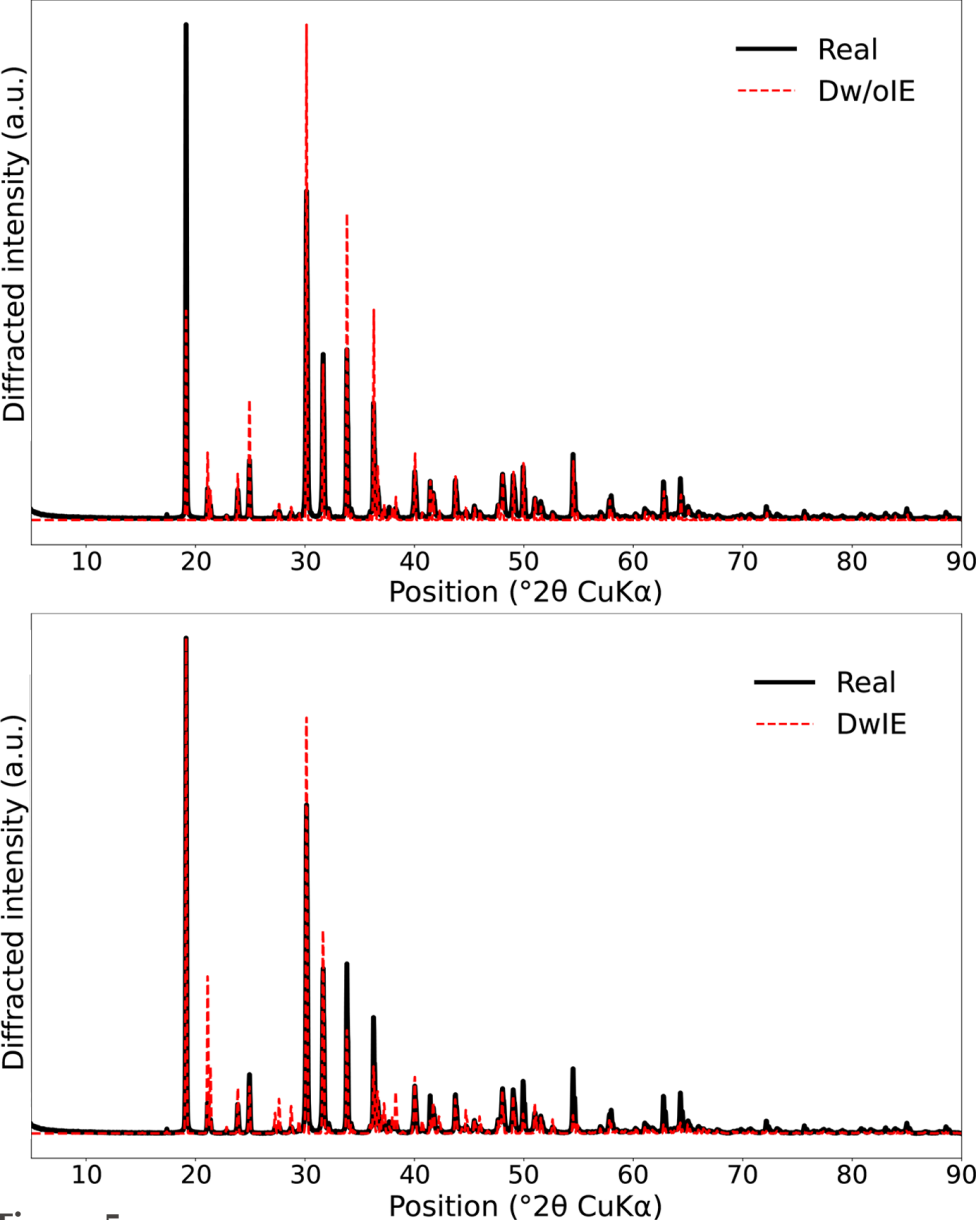
Comparison between real and simulated data from both databases (DwIE and Dw/oIE). The experimental XRD pattern is a mixture of the four mineral phases with abundances of around 40% calcite, 20% gibbsite, 20% dolomite, 20% hematite (Sample 20 in Table 1 ▸).

Thus, the expression of the corrected one-dimensional signal 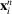 is
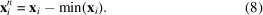
An example of simulations from each database is displayed in Fig. 5 ▸.

### 2.4. Neural network training and validation set

To monitor the network training, at each epoch (*i.e.* each time run through the training set), the method is evaluated using an independent set termed the ‘validation set’ and denoted 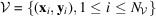. Using this set allowed us to track the evolution of evaluation metrics over epochs on a dataset independent from the training set. This provided insights into the effectiveness of the training and the possibility of overfitting to the training data. As previously mentioned, the validation set contained 2500 synthetic data. The method was evaluated using three metrics to measure the error between predictions 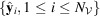 and the ground truth **y**_*i*_.

**Figure 6:**
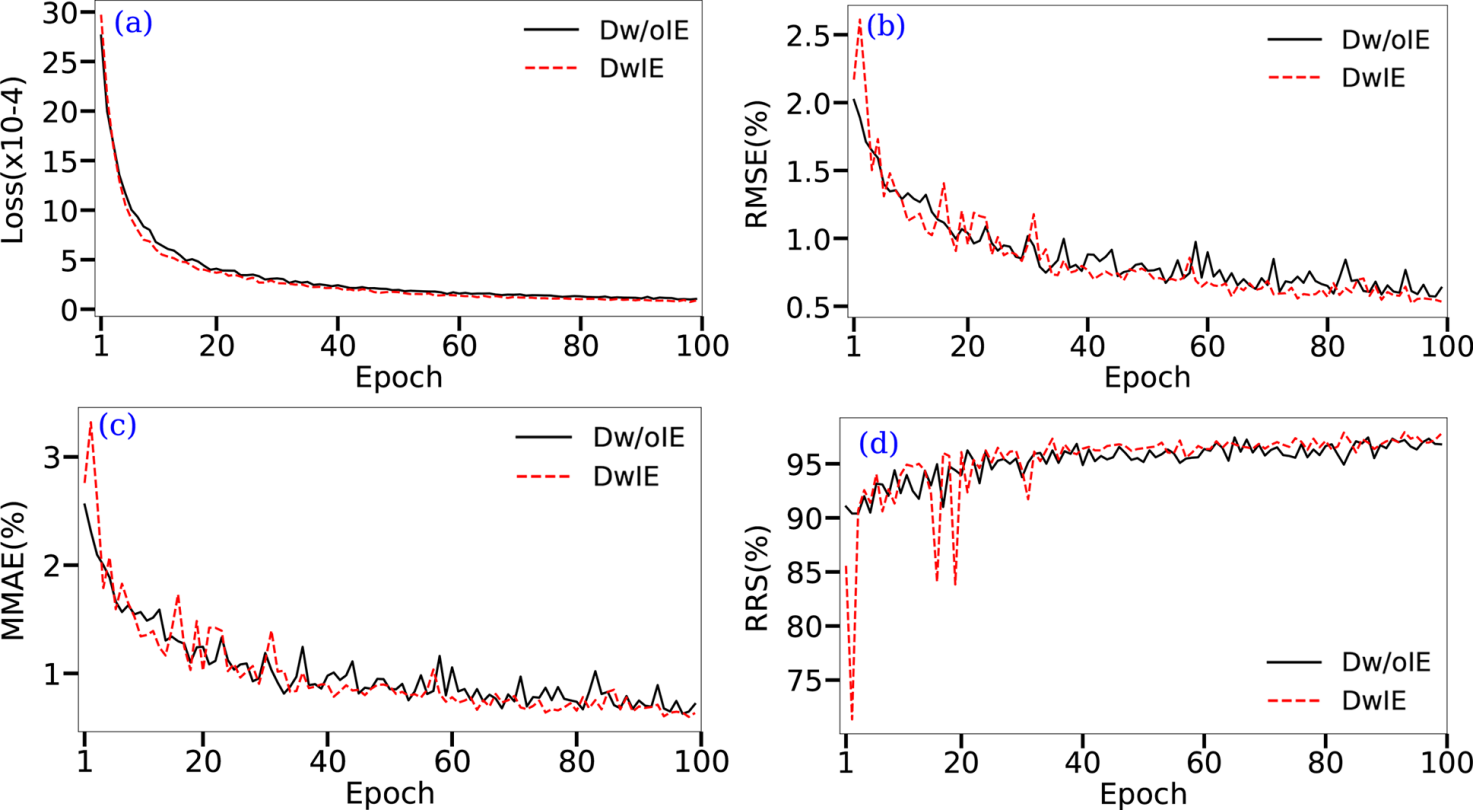
Monitoring of NN training for each database (DwIE and Dw/oIE): (*a*) training loss evolution for each training epoch; and RMSE (*b*), MMAE (*c*) and RRS (*d*) measures on the validation set at each epoch.

**Figure 7:**
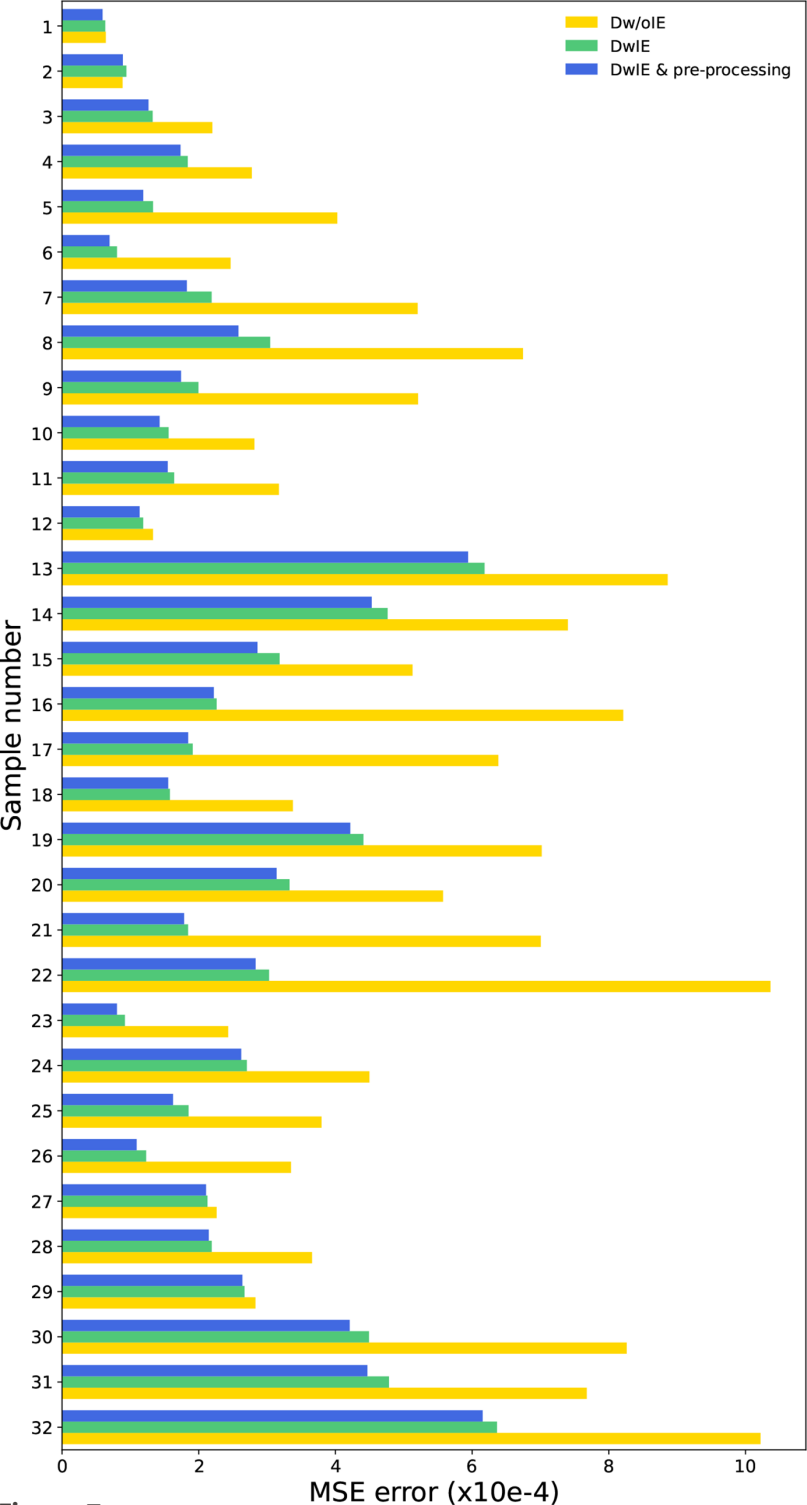
MSE comparison between the 32 real data and synthetic data. Blue for Dw/oIE, red for DwIE, gray for DwIE with real data pre-processing (see Section 2.3.2). The simulations have the same mineral phases proportions as the experimental XRD patterns, and the parameters are optimized using the Rietveld refinement.

First, the root mean square error compares **y**_*i*_ and 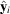, and is defined as follows: 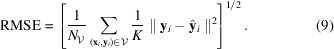
An alternative way to compare *y*_*i*_ and 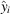 is the mean maximal absolute error (MMAE), expressed as follows: 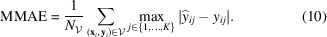
MMAE can be interpreted as a percentage accuracy on all the inferred proportions. The last measure used in this paper is the rate of recovered support (RRS), which quantifies the capacity of the network to correctly identify components by comparing true components with predicted ones. However, due to Dirichlet modeling, the components of the CNN prediction (*i.e.***y**_*ij*_) are not exactly equal to zero. Thus, the predicted support is defined with a low-value threshold ɛ. Given an array **y** ∈ Δ_*K*_ and ɛ ∈ (0, 1), we define 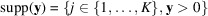
and 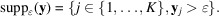
The RRS is defined as 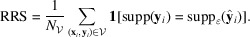
In the following, two threshold values were considered. The first was arbitrarily set to ɛ = 0.01, and the second was adapted as a function of the MMAE value. To summarize, MMAE and RMSE serve to measure the quantification quality, while RRS is employed for identification. All three metrics are also used in Table 2 ▸ to highlight the need for a large amount of training data to ensure successful CNN training. As mentioned above, this clearly shows that even training a CNN with 1000 data points leads to poor mineral phase identification and quantification. However, obtaining a sufficient number of experimental XRD patterns with the required variability is not feasible in practice. This makes it necessary to resort to synthetic data for training the network. Based on our experiments (see Table 2 ▸), a training set of 10 000 appears to be sufficient to address the problem of mineral phase quantification. The evolution of the epoch loss and of the three metrics (MMAE, RMSE and RRS) as a function of the epoch, on the validation set and during the training, is provided in Fig. 6 ▸. This allows us to assess the number of epochs required to train the CNN. For the databases presented in Section 2.3.2, a sharp decrease in the loss over the first 40 epochs is observed. Then, the decrease in loss continues, but at a slower rate, and finally stabilizes during the last ∼10 epochs. This suggests that training the network for more than 100 epochs does not bring significant improvements in the presently used CNN configuration, *i.e.* with a constant learning rate. This is supported by the fact that, for both databases, the evolution of the RMSE and MMAE is similar to the epoch loss, while the RRS also plateaus in the last ∼10 epochs (Fig. 7 ▸). Even in the early epochs, the CNN proves effective, reaching an RMSE of around 1.5% after 10 epochs. However, as will be shown later, using a learning rate scheduler over a larger number of epochs allows the loss to continue decreasing concomitantly with the error metrics.

Overall, RMSE and MMAE quickly reach values of around 5%, while RRS approaches 85%. The evolution of measurements on the validation set shows that CNN training is not subject to overfitting with training data, as shown by the consistent decrease in the validation errors. In the next section, for both databases the training with the smallest MMAE on the validation set will be considered the best training.

## 3. Results

Unless otherwise mentioned, all results presented in this section were obtained with one of the 10 000 synthetic XRD pattern training sets.

### 3.1. Simulated test set

The efficiency of the proposed approach was first evaluated with our test set of 2500 synthetic XRD patterns, which is independent of both training and validation sets. For all our experiments, we did not observe any performance difference between the validation set and the test set.

Table 3 ▸ presents the mean and standard deviation across five training runs for each database. This serves as a measure of method stability. First, for the Dw/oIE dataset, the mean RMSE of the five training sets was 10.44 ± 12.07% (mean ± standard deviation). Among the five training sessions conducted, three were successful. For these three successful training sets, the mean RMSE was 0.58 ± 0.00%. For the DwIE dataset, four training sets out of five were successful. The mean RMSE of the five training sets was 5.49 ± 9.98%, while that of the four successful was 0.50 ± 0.02%. For both datasets, the high proportion of successful training sets (60–80%) highlights a satisfying stability.

For the successful training sets, the MMAE, RMSE and RRS of the Dw/oIE were 0.65 ± 0.20%, 0.58 ± 0.00% and 97.01 ± 0.24%, respectively, hence demonstrating the ability of the CNN to identify and quantify the different minerals. However, introducing the instrumental effects into the calculation (DwIE training) significantly improved the quality of the predictions, with an absolute decrease in the RMSE and MMAE parameters of 0.08 and 0.07%, respectively, and a 0.21% increase in the RRS value. These observations hold true for the best training, with comparable yet systematically better values for the Dw/IE training compared with the Dw/oIE training (Table 3 ▸). Note that no comparison with the Rietveld refinement (Rietveld, 1969 ▸) (detailed below in Section 3.2) was done, because the equations used here for generating synthetic patterns are similar to those minimized by the Rietveld method. Hence, the method used for producing data and for minimizing errors during quantification would have been correlated, and calculation of a residual error meaningless.

### 3.2. Experimental XRD patterns

**Figure 8:**
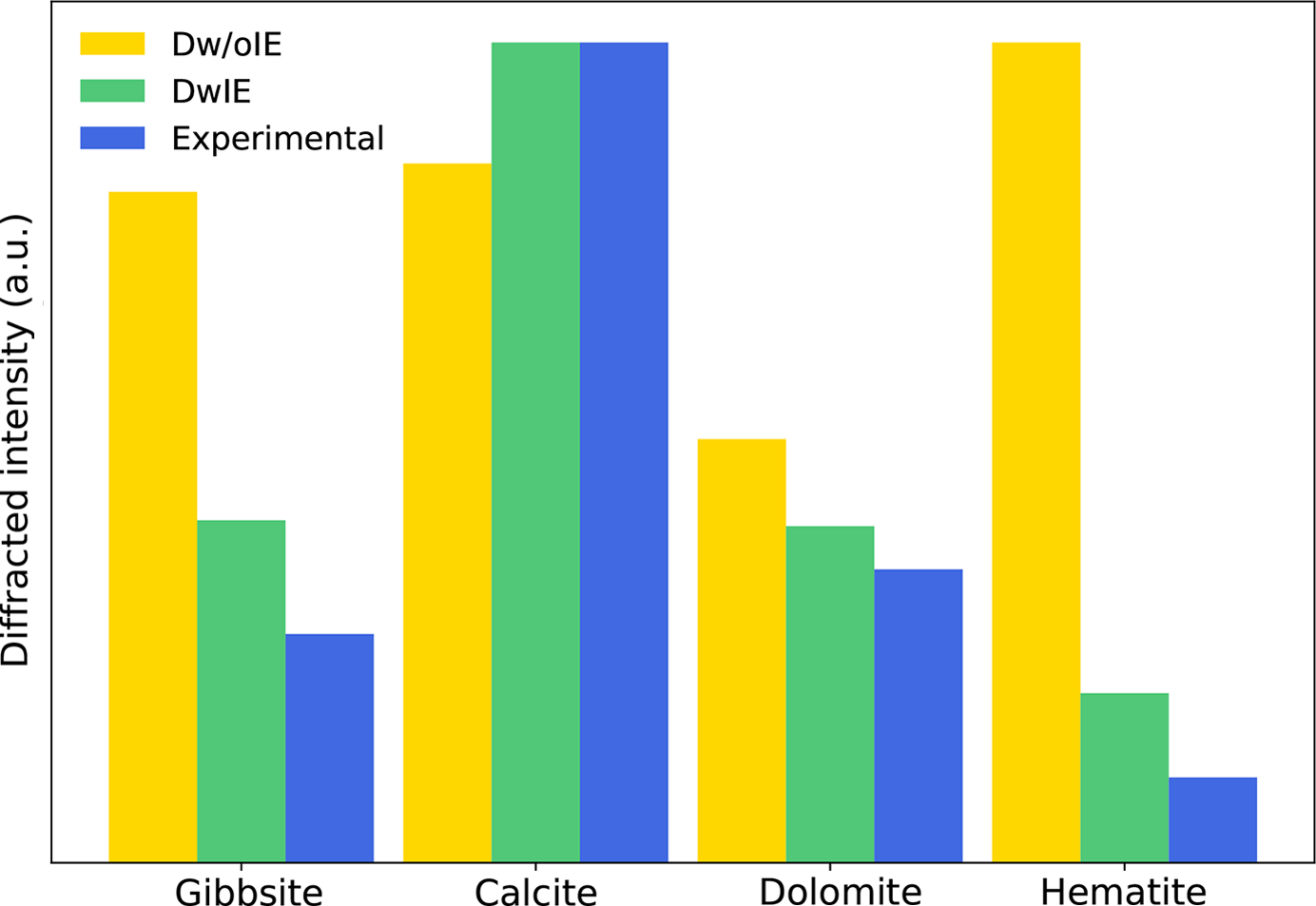
Absolute diffracted intensity for the four mineral phases.

In this section, an experimental XRD database consisting of 32 patterns and specifically acquired for this study was used to evaluate the robustness of the presently proposed approach to the quantification of not only simulated data, but also real data. Although relatively small, this evaluation provides valuable insights into the efficiency of the method. These experimental XRD patterns were recorded on pure minerals and on assemblages that are mixtures of the same mineral phases as in the synthetic dataset (see Table 1 ▸). Fig. 8 ▸ compares the maximum value of the diffracted intensity for the four mineral phases from the DwIE, the Dw/oIE and the experimental databases, thus highlighting the importance of including instrumental effects in the simulation to be closer to real XRD patterns.

**Figure 9:**
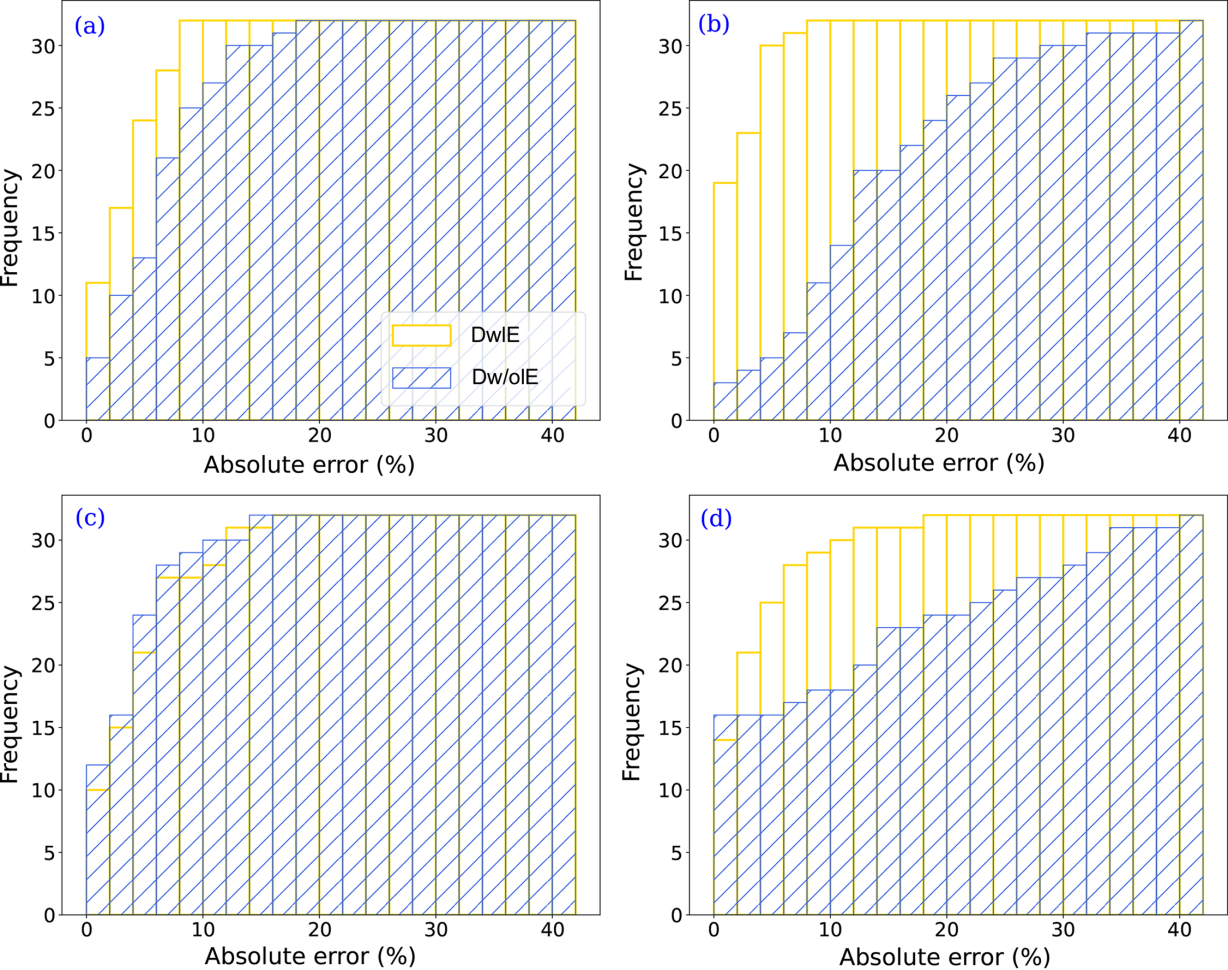
Cumulative histograms of absolute error by classes for the 32 experimental XRD patterns: (*a*) calcite, (*b*) gibbsite, (*c*) dolomite and (*d*) hematite. Each bar of the histogram corresponds to a interval of 2%, from 0 to 40%.

Fig. 7 ▸ compares the mean square error between synthetic and experimental XRD patterns for Dw/oIE and DwIE databases, while Table 4 ▸ provides the results for both training sets. The training sets that performed poorly on synthetic databases also yielded unsatisfying results on the experimental data. However, compared with the simulated datasets, the difference between the Dw/oIE and DwIE databases was more marked, with the DwIE being ∼10% more efficient in terms of MMAE and RMSE. Whereas Dw/oIE was 20% more efficient in terms of RRS. The Dw/oIE performance for RRS, although not intuitive, is probably related to its absence of the consideration of the linear attenuation factor. This leads to a more discernible signal for the phases present in low concentration and hence better identification (less difference in maximum intensity between the different phases). In turn, this biases the quantification because of inaccurate intensity ratios between the different phases, thus explaining the higher MMAE and RMSE values. To assess the validity of the hypotheses, cumulative frequency histograms, based on absolute errors for each class, are plotted in Fig. 9 ▸ that compare the DwIE and Dw/oIE database with cumulative histograms (one for each mineral phase). The most efficient database is the one which reaches its maximum (here the maximum number of experimental XRD patterns = 32) the fastest. Even with smaller values of RRS, the DwIE performed better, based on absolute errors. This highlights that the chosen RRS threshold value has a major influence for comparison of different training sets, and must be considered with care. To minimize such threshold effects, two different RRS values were calculated, with threshold values of 1 and 7%, with the second threshold value being chosen according to the MMAE. This approach enabled us to focus on prediction errors rather than small quantification errors. For the DwIE database, the RRS increased from 43.75 to 71.87% with an adapted RRS threshold. This indicates that the majority of prediction errors have small values, highlighting the efficiency of the DwIE database for training.

The substantial differences in RRS between synthetic and experimental XRD patterns likely stems from the complexity of the experimental data, which contain not only pure diffraction data, but also for example instrumental effects, ‘noise’ related to detector sensitivity and accuracy, air scattering *etc.* While describing accurately how the NN discerns information from the data remains challenging, it may be speculated that some low-intensity background ‘peaks’ related to, for example, statistical errors of count may be interpreted by the NN as actual diffraction peaks. Consequently, the algorithm may attribute small proportions to certain mineral phases, hence contributing to the observed disparity in RRS.

**Figure 10:**
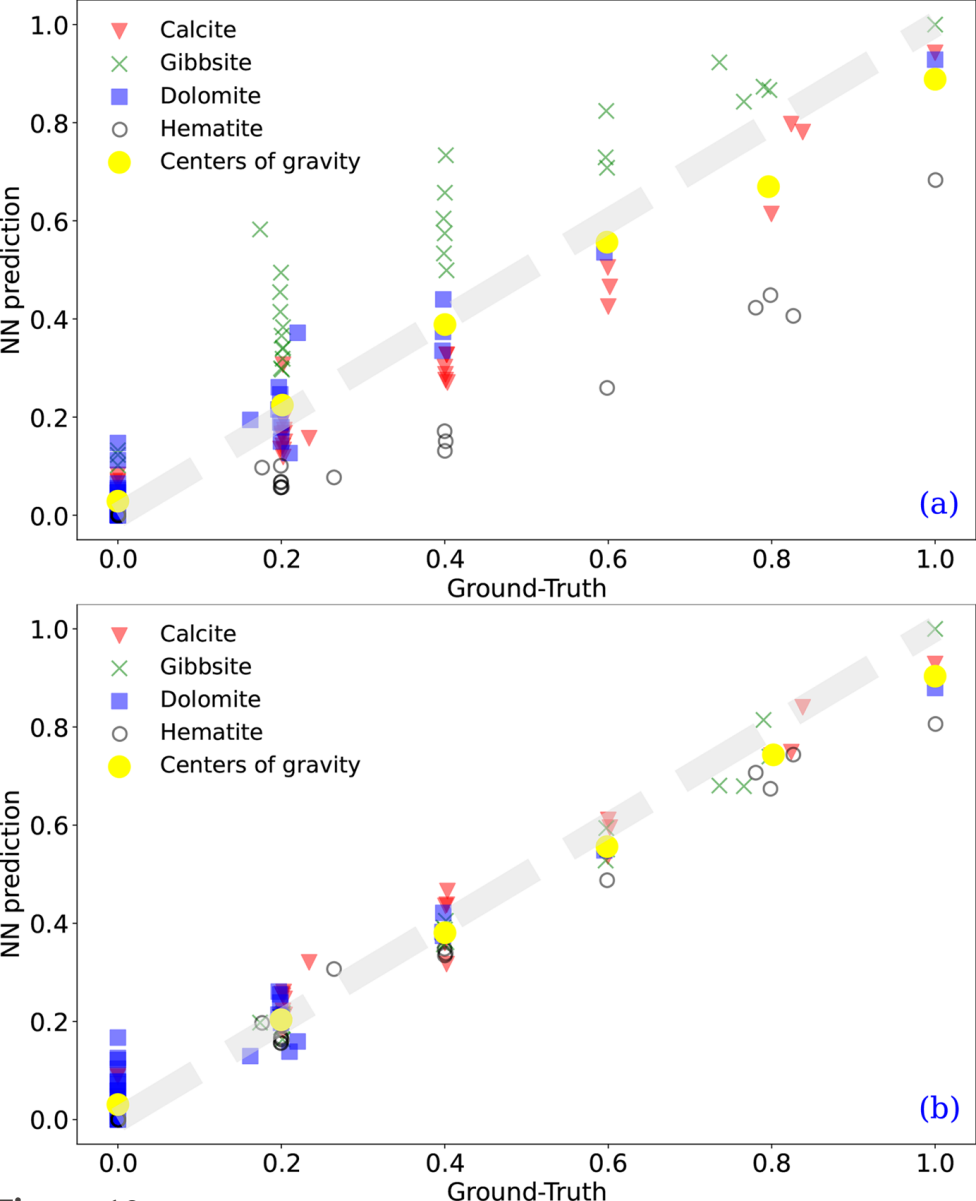
Experimental test set: scatter plot of the prediction according to the ground truth: (*a*) trained with Dw/oIE, (*b*) trained with DwIE.

Overall, these results show that taking into account the instrumental effects on the XRD patterns (DwIE training) improves the quality of both phase identification and quantification compared with the NN method trained from pure XRD profiles (Dw/oIE training). Real data are affected by uncertainties arising from instrumental parameters (*e.g.* detector efficiency, source brightness and divergence). Consequently, analyzing real data is obviously more challenging than working with simulated data, and this discrepancy certainly accounts for the performance gap. Fig. 10 ▸ illustrates the importance of simulation quality by comparing the performance of both databases (DwIE and Dw/oIE).

In terms of performance, the DwIE training database yielded very good results with an RMSE value of 5.16% and MMAE of 6.96%. Fig. 10 ▸ illustrates the predictions compared with the ground truth for this training. Each point corresponds to one of the 32 four-dimensional proportion vectors of the experimental XRD pattern. The center of gravity and the majority of the prediction points align with the identity function, indicating good predictions. Interestingly, the centers of gravity obtained with the DwIE or Dw/oIE training are comparable, but data scattering is much lower with the DwIE training, and systematic quantification biases were only observed in the Dw/oIE training, where hematite is underestimated and gibbsite is overestimated. Because of the high scattering observed with this training database [Fig. 10 ▸(*a*)], increasing the RRS threshold from 1 to 7% led to no improvement in the RRS value (75%, see Table 4 ▸).

Not surprisingly, the Rietveld refinement outperformed our methods, since the network was exclusively trained on simulated data, while Rietveld refinement incorporates slightly more refinement parameters and aims to minimize the RMSE. Indeed, with respect to the RMSE, the Rietveld method achieves a precision of 1.27%. In contrast, our method presents a comparable RMSE that is only 4% higher. This further underscores the value of a CNN based method for XRD analysis, or alternatively, a hybrid analysis combining both methodologies.

## 4. Discussion and perspectives

Here, an automated analysis method for XRD patterns was proposed. It proved capable of both identifying and quantifying mineral phases within a material sample. Utilizing an NN and optimizing a specially designed loss function for proportion inference, the proposed method, which is a two-step approach described by Fig. 1 ▸, demonstrated robust performance. A main aspect of our strategy lay in the training phase of the NN, which exclusively employs synthetic data. This allowed us to generate a wide variety of XRD patterns, both for a given phase, where the structural and size parameters were varied (intra-class variation), and for mixtures of mineral phases. Consequently, this approach not only enhances performance but also facilitates the analysis of extensive experimental databases. A very important aspect of the proposed method is that, in contrast to the Rietveld refinement, no human intervention is needed to identify phases before the quantification. A database containing a sufficient number of different mineral phases should be able to find, with good precision, the abundance of each phase in multiphase compounds. Furthermore, our method excels in terms of analysis of speed once the NN is trained. These collective advantages open the door to the identification and quantification of mineral phases within large datasets, such as time-resolved synchrotron analyses or XRD-CT data. Datasets of this nature are typically composed of up to ten mineral phases. Thus, to keep the same accuracy, the number of training data must be increased, leading to increased calculation time. To maintain a constant ratio of the number of data in the test set relative to the total number of data, accounting for variations in lattice parameters and proportion vectors sampled, an exponential increase in the number of XRD patterns calculations should be done. However, based on our knowledge, a linear augmentation should be enough to achieve similar performance.

Further improvements in the calculation of synthetic data could consist of considering counting statistics, or employing explicit calculations of XRD patterns, for example using numerical methods proposed by Debye (1915 ▸), Warren (1990 ▸), or Drits & Tchoubar (2012 ▸). Specifically, these methods allow us to calculate the actual profile of each diffraction maximum instead of using a Gaussian intensity distribution, as done in the present work. This approach offers significant advantages, particularly when dealing with XRD patterns of anisomorphic mineral phases: a common occurrence in XRD-CT data. Another aspect to explore is the database construction (*i.e.* the distribution of single-phase and multicompound XRD patterns) as well as the proportion distribution.

**Figure 11:**
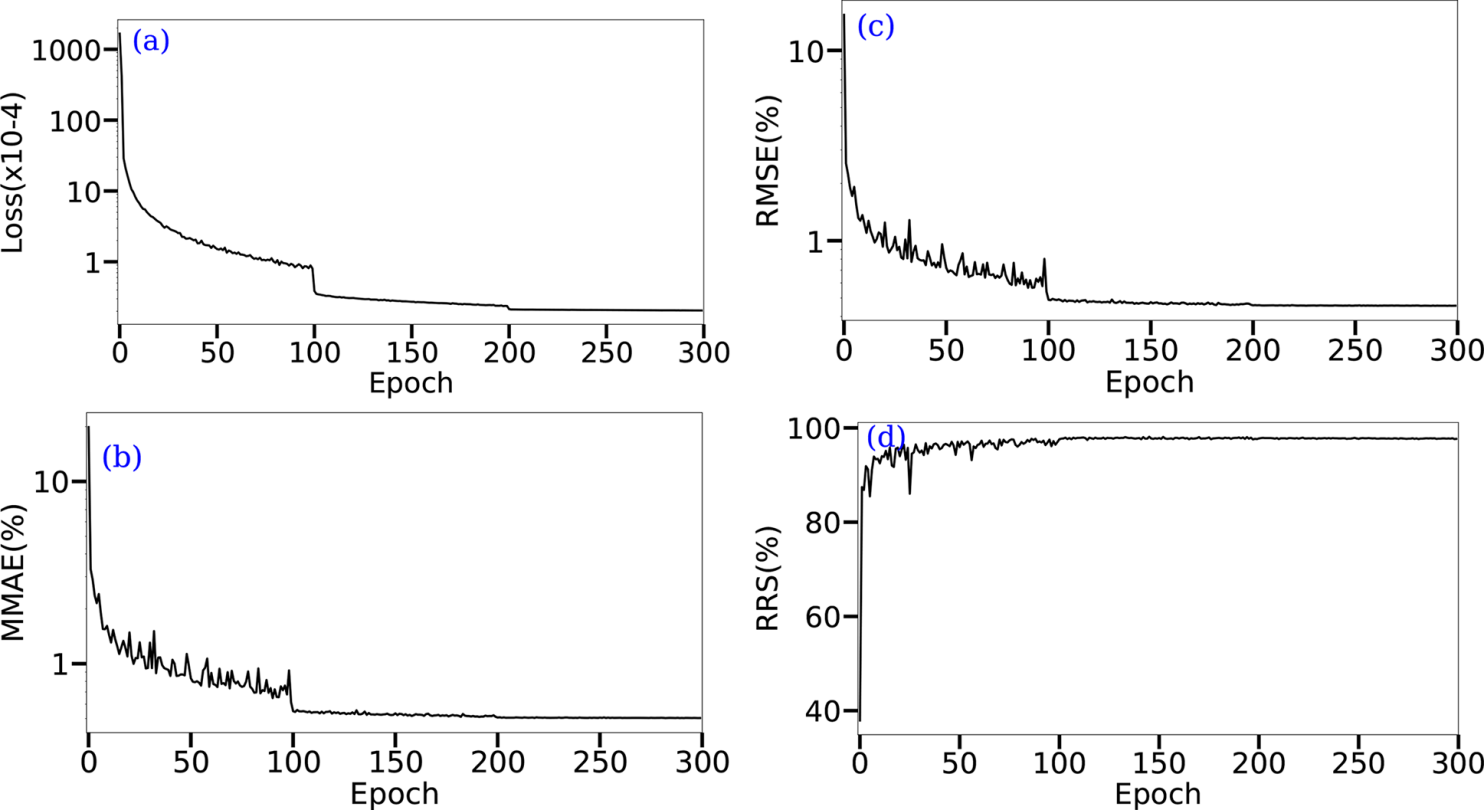
Monitoring of NN training for the DwIE database. In total, 300 epochs were performed with a learning rate scheduler (0–100, lr = 1*e* − 3, 101–200, lr = 1*e* − 4 and 201–300, lr = 1*e* − 5). (*a*) Training loss evolution for each training epoch, (*b*) RMSE, (*c*) MMAE and (*d*) RRS measures on the validation set at each epoch. (*a*), (*b*) and (*c*) are presented with a logarithmic scale.

Additional enhancements can be made at the NN level. First, regarding the choice of network architecture, particularly the hidden layers of the network, the number of layers, their dimensions and the type of layers can be discussed (Goodfellow *et al.*, 2016 ▸). Optimization can also be achieved by selecting the best activation functions between each of these layers (Sharma *et al.*, 2020 ▸). Additionally, various optimization hyperparameters can be adjusted, such as the optimizer (Choi *et al.*, 2019 ▸), the number of epochs, batch size and learning rate (Smith, 2018 ▸). To test such effects, an experiment where a network was trained with 300 epochs was carried out, in which a learning rate scheduler (Fig. 11 ▸) was used. Interestingly, the epoch loss decreased after 100 epochs, *i.e.* when the learning rate was reduced. Additionally, the error metrics on the validation set decreased, further highlighting the benefits of a learning rate scheduler. For the experimental test set it enhanced the results across all metrics by approximately 10%. Specifically, the RMSE was reduced to 4.75%, the MMAE to 6.21% and the RRS_7%_ to 87.5%. Otherwise, further investigation into the application of auto-encoders (or the Unet architecture) should be considered for future work. Such an extension would explore the reconstruction aspects of XRD patterns, enabling a comparison between the original and reconstructed signals. This comparison can serve in the identification of missed peaks, potentially corresponding to unknown mineral phases.

## Figures and Tables

**Table 1:** Composition of the 32 experimental datasets in terms of fraction of mineral phases

Data	Calcite	Gibbsite	Dolomite	Hematite
Sample 1	0	1	0	0
Sample 2	0.203	0.797	0	0
Sample 3	0.403	0.597	0	0
Sample 4	0.600	0.400	0	0
Sample 5	0.800	0.200	0	0
Sample 6	1	0	0	0
Sample 7	0.204	0.2001	0.595	0
Sample 8	0.402	0.200	0.398	0
Sample 9	0.602	0.201	0.196	0
Sample 10	0.201	0.402	0.397	0
Sample 11	0.401	0.399	0.200	0
Sample 12	0.202	0.599	0.199	0
Sample 13	0.202	0.200	0	0.598
Sample 14	0.402	0.198	0	0.399
Sample 15	0.599	0.201	0	0.199
Sample 16	0.199	0.401	0	0.400
Sample 17	0.401	0.400	0	0.199
Sample 18	0.202	0.597	0	0.200
Sample 19	0.204	0.199	0.197	0.401
Sample 20	0.400	0.202	0.199	0.199
Sample 21	0.204	0.398	0.199	0.199
Sample 22	0.201	0.202	0.398	0.199
Sample 23	0	0	1	0
Sample 24	0	0	0	1
Sample 25	0.824	0	0	0.176
Sample 26	0.838	0	0.162	0
Sample 27	0.234	0.766	0	0
Sample 28	0	0.736	0	0.264
Sample 29	0	0.790	0.210	0
Sample 30	0.202	0	0	0.798
Sample 31	0	0	0.220	0.78
Sample 32	0	0.174	0	0.826

**Table 2:** Performance comparison of a trained NN on the experimental test set Each network is trained using the DwIE training set from 100 to 100 000 XRD patterns. The calculation time remains constant.

Training set size	Epochs	RMSE	MMAE	RRS_1%_
100	10000	34.14	46.69	12.5
1000	1000	45.22	68.42	16.67
10000	100	**5.16**	6.96	43.75
**100000**	10	5.84	**6.68**	**51.52**

**Table 3:** RMSE, MMAE and RRS values (%) for the synthetic test set Top (first three rows): mean value and standard deviation over five training sessions; middle (next three rows): mean value and standard deviation for the successful training; bottom (last three rows): mean value and standard deviation for the best training.

	RMSE ↓	MMAE ↓	RRS_1%_ ↑
	5 training sets		
Dw/oIE	10.44% ± 12.07 1	4.46% ± 16.91	70.16% ± 32.89
**DwIE**	**05.49%** ± **09.98**	**07.58%** ± **14.00**	**83.49%** ± **27.49**
	Successful training sets		
Dw/oIE	00.58% ± 00.00	00.65% ± 00.20	97.01% ± 00.24
**DwIE**	**00.50%** ± **00.02**	**00.58%** ± **00.03**	**97.22%** ± **00.47**
	Best training sets		
Dw/oIE	0.58%	0.62%	97.20%
**DwIE**	**0.49%**	**0.55%**	**97.40%**

**Table 4:** RMSE, MMAE and RRS values (%) for experimental test set Top (first three rows): mean value and standard deviation over five training sessions; middle (next three rows): mean value and standard deviation for the successful training and (next three rows) mean value and standard deviation for the best training; bottom (last row): result obtained using a Rietveld refinement to the data.

	RMSE ↓	MMAE ↓	RRS_1%_ ↑	RRS_7%_ ↑
	5 training sets			
Dw/oIE	18.79% ± 07.15	26.92% ± 12.06	**45.62%** ± **27.36**	49.09 ± 30.33
**DwIE**	**09.37%** ± **09.12**	**13.51%** ± **14.12**	38.75% ± 13.35	**65.62%** ± **27.67**
	Successful training sets			
Dw/oIE	12.96% ± 00.24	17.08% ± 00.36	**67.71%** ± **05.31**	75% ± 02.55
**DwIE**	**04.82%** ± **00.74**	**06.47%** ± **01.15**	45.31% ± 02.71	**78.91%** ± **08.71**
	Best training			
Dw/oIE	12.71%	16.68%	**75.00%**	**75.00%**
DwIE	**05.16%**	**06.96%**	43.75%	71.87%
**Rietveld**	**01.27%**	**01.78%**	**100%**	**100%**

## Data Availability

The Python code developed for XRD pattern simulation is available on GitHub (https://github.com/titouansimonnet/XRD_Proportion_Inference). To read the CIF, the code uses the Python package *Crystals* (René de Cotret *et al.*, 2018 ▸).
